# Urinary L-type fatty acid-binding protein is a predictor of cisplatin-induced acute kidney injury

**DOI:** 10.1186/s12882-022-02760-4

**Published:** 2022-03-31

**Authors:** Masaaki Yanishi, Hidefumi Kinoshita

**Affiliations:** Department of Urology and Andrology, Graduate School of Medicine, Kansai Medical University, 2-5-1 Shinmachi, Hirakata, Osaka, 573-1010 Japan

**Keywords:** L-type fatty acid-binding protein, Acute kidney injury, Cisplatin, Urinary biomarker, Renal function

## Abstract

**Background:**

Although cisplatin-based chemotherapy is a standard treatment for urothelial carcinoma, it often causes acute kidney injury (AKI). AKI and dysfunction are observed in 25–35% of cisplatin-based chemotherapy patients, who may require treatment down-titration or withdrawal. In this study, we evaluated whether urinary L-FABP is a marker for early diagnosis of cisplatin-caused AKI.

**Methods:**

We included 42 adult patients who underwent cisplatin-based chemotherapy for bladder cancer or upper tract urothelial carcinoma from January 2018 to March 2019. Urinary L-FABP and serum creatinine were measured at 2 and 6 h, and 1, 2, 3, 7 and 28 days after taking cisplatin.

**Results:**

In the first week after receiving cisplatin, 10 patients (23.8%) were diagnosed with AKI (AKI^+^ group). Pre-treatment (baseline) measurements did not significantly differ between the AKI^+^ and AKI^−^ groups. However, urinary L-FABP concentrations rapidly increased in the AKI^+^ group and were significantly greater than in the AKI^−^ group at Hour 2, Hour 6, Day 1 and Day 2. Serum creatinine also significantly differed between the AKI^+^ group and the AKI^−^ group on Days 3 and 7. ROC analysis was performed to evaluate the superiority of urinary L-FABP magnification which had the highest at the hour 6. The urinary L-FABP magnification and levels of aria under curve was 0.977. Based on ROC analysis, the best cut-off value of urinary L-FABP magnification was 10.28 times urinary L-FABP levels at the hour 0 (base line urinary L-FABP).

**Conclusions:**

Acute renal function deterioration was predicted by increased urinary L-FABP excretion within 6 h after receiving CIS-CT and, in those with AKI, the increase in urinary L-FABP excretion preceded the rise in sCr by over 2 days. In contrast, no appreciable changes in urinary L-FABP levels were observed in patients with stable renal function throughout the whole observation period. So early increase in urinary L-FABP may identify patients at risk of cisplatin-induced AKI, who might benefit from treatment to prevent nephrotoxicity.

**Trial registration:**

This study was retrospectively registered.

## Background

Although cisplatin-based chemotherapy (CIS-CT) is a standard treatment for urothelial carcinoma, it often causes nephrotoxicity. Acute kidney injury (AKI) and dysfunction are observed in 25–35% of CIS-CT patients, who may require treatment down-titration or withdrawal [1, 2]. Cisplatin predominantly accumulates in, and is excreted through, the kidneys. Concentrations that are nontoxic in the blood may be toxic in the kidneys, because concentrations in the tubule epithelial cells are five times higher than in blood. Increasing drug dosage is limited by dose-dependent renal toxicity, which may compromise treatment effectiveness. Toxic effects occur primarily in the proximal tubules, particularly in tubule epithelium cells on segment S-3. This is likely to be the structural basis for the severe reduction in kidney perfusion and glomerular filtration rate (GFR), seen within 48–72 h of receiving CIS-CT [3–5]. Current AKI diagnostic criteria include increased serum creatinine (sCr) [6]. However, among patients with acute renal insufficiency, accurate estimations of the timing and severity of injury cannot be reliably derived from concomitant changes in sCr and GFR, which may result in delayed diagnosis and intervention, and underestimation of injury severity [7]. Large and rapid changes in GFR may be paralleled by relatively small changes in sCr, in particular in early-phase kidney injury [8]. Several AKI biomarkers have shown relevance in revealing kidney injury, especially in septic patients, subjects in critical condition, heart surgery patients, and in individuals with contrast-induced nephropathy. The most commonly studied markers are neutrophil gelatinase-associated lipocalin (NGAL), interleukin-18 (IL-18), kidney injury molecule-1 (KIM-1), L-type fatty acid-binding proteins (L-FABP), and cystatin C [1, 9, 10], all of which are associated with improved early kidney injury detection, compared with traditional tests based on creatinine and GFR.

Because acute tubular necrosis is the common feature of most AKI, predicting histological injuries in renal tubular cells would be a required characteristic of renal biomarkers for AKI. For AKI in patients who had undergone cardiac surgery, urinary L-FABP levels had increased within 4 h after surgery in AKI patients, whereas sCr started to increase 48 h later [11]. We recently reported that urinary L-FABP peaked within 2 h of declamping in laparoscopic partial nephrectomies, and suggested that urinary L-FABP can help predict the extent of ischemic renal injury [12]. In this study, we examined whether urinary L-FABP is a marker for early diagnosis of AKI from CIS-CT.

## Materials and methods

This study cohort included 42 patients, aged > 20 years, who received CIS-CT for histologically diagnosed bladder cancer or upper tract urothelial carcinoma at Kansai Medical University Hospital, from January 2018 to March 2019. Each patient had an Eastern Cooperative Oncology Group performance status of 0 or 1, and had an estimated GFR over 45 ml/min. Kidney function was evaluated on the basis of estimated GFR, which was calculated from sCr concentrations using the standard conversion formula for Japanese individuals [13].

All patients provided written informed consent. The Institutional Review Board of Kansai Medical University (IRB approval number: H1602120) approved the study protocol, which was performed in accordance with the Declaration of Helsinki.

Baseline measurements of sCr and urinary L-FABP were performed the day before receiving CIS-CT. On the next day, just before CIS-CT administration, each patient received an infusion of 2000 ml of isotonic electrolyte solution (8.1 mEq magnesium sulphate; KCl 15% 10 mEq; mannitol 150 ml (20%) and dexamethasone. Next, they each received a 2-h intravenous infusion of 70 mg/m^2^ of cisplatin followed by 500 ml of a 5% glucose solution and 500 ml of lactated Ringer’s solution. Urinary L-FABP and sCr were measured at Hours 2 and 6 and Days 1, 2, 3, 7 and 28 after receiving cisplatin. Urinary L-FABP concentrations were measured by enzyme-linked immunosorbent assay using the Human L-FABP ELISA Kit (CMIC, Tokyo, Japan). As the urinary L-FABP value varied for each patient, we calculated magnifications from the pre-treatment (baseline) value and compared them:

Magnification = [Post-treatment urinary L-FABP value {i.e., at Hours 2 and 6 and Days 1, 2, 3, 7 and 28}]/ [Pre-treatment urinary L-FABP value].

To diagnose AKI, we used the specific criteria introduced by 2012 Kidney Disease: Improving Global Outcomes (KDIGO) Acute Kidney Injury Work Group [14]. AKI was defined as (a) sCr elevation exceeding 0.3 mg/dL within 48 h; (b) increase in sCr to > 1.5 times the baseline value (obtained within the previous 7 days); or (c) urine volume ≤ 0.5 mL/kg/hour for 6 h.

Data are presented as mean ± standard deviation. Differences in continuous variables were compared with the Mann–Whitney U test. A *p* value of < 0.05 was considered statistically significant. The best cut-off point for urinary L-FABP levels were determined using receiver operating characteristic (ROC) analysis. All statistical analyses were performed using Stat-View for Windows statistical software (Abacus Concepts Inc., Berkeley, CA, USA).

## Results

During the first week after receiving CIS-CT, 10 of the patients (23.8%) were diagnosed with AKI (AKI^+^ group). Among these 10 patients, 5 had elevated sCr levels (> 0.3 mg/dL within 48 h), 5 had increased sCr levels (> 1.5 × baseline value), and 2 had low urine volumes (≤ 0.5 mL/kg/h for 6 h). These 10 patients comprised the AKI^+^ group. The 32 patients without appreciable changes in sCr throughout the observation period comprised the AKI^−^ group. All patients had complete serial serum and urinary L-FABP evaluations.

Characteristics of the AKI^+^ and AKI^−^ groups are shown in Table 1. The two groups did not significantly differ in age, BMI or kidney function, but there were significantly more women in the AKI^+^ group (*P* = 0.046).

**Table 1:**
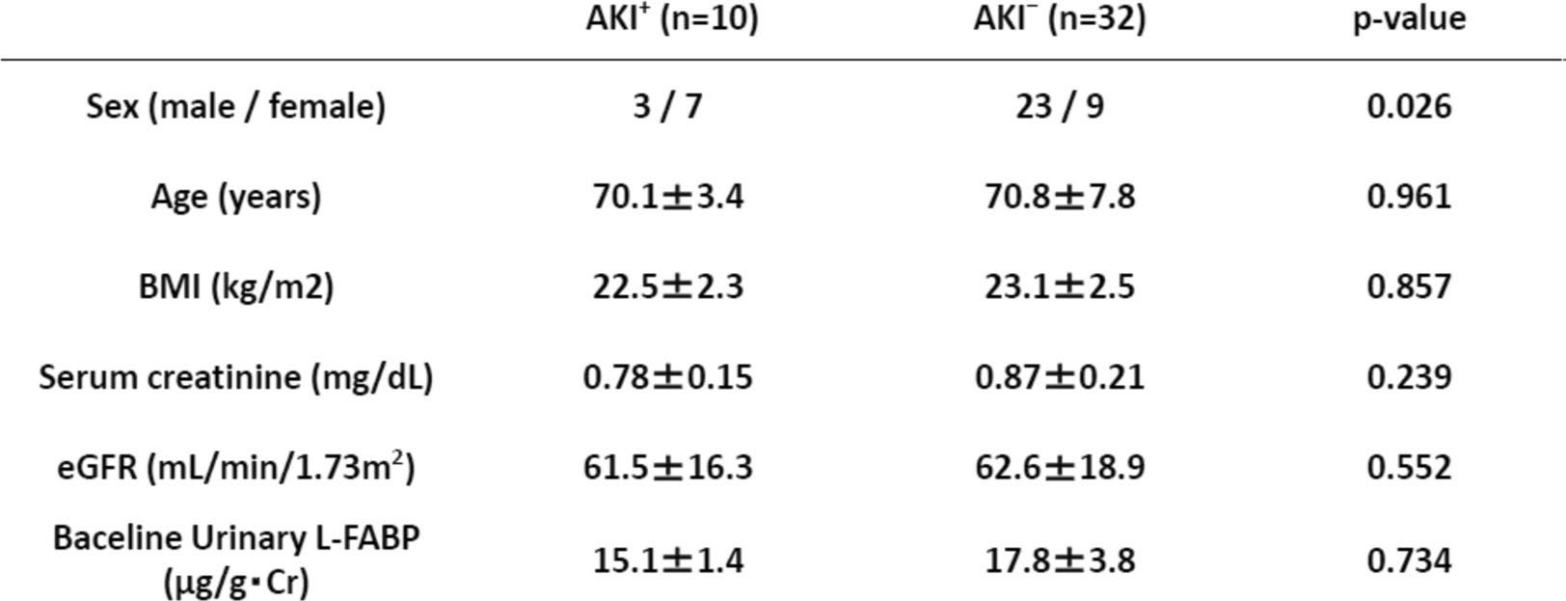
Patients characteristic of AKI^+^ and AKI^−^ groups

**Fig. 1:**
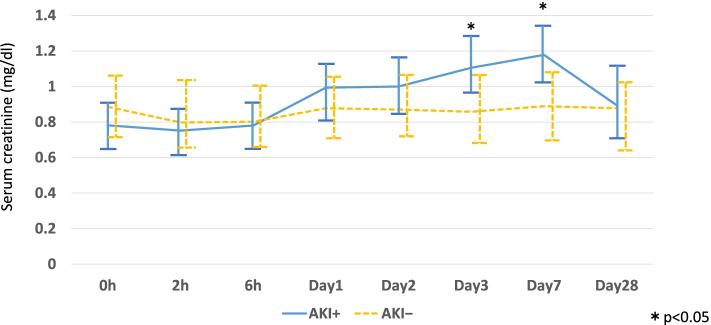
Comparison of changes in serum creatinine levels between AKI^+^ and AKI^−^ groups

Changes in post-treatment sCr over time in the AKI^+^ and AKI^−^ groups are shown in Fig. 1. Although sCr levels did not significantly differ between the two groups immediately after receiving CIS-CT, sCr changes were significantly different between the two groups by Days 3 and 7.

**Fig. 2:**
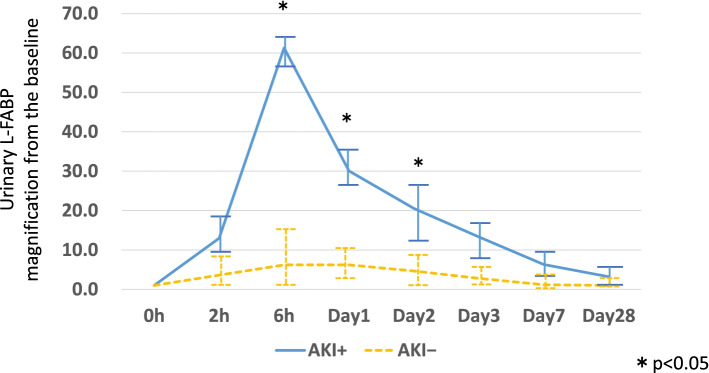
The changes of urinary L-FABP magnification over time in the AKI^+^ and AKI^−^ groups

Figure 2 shows changes in urinary L-FABP magnification over time in the AKI^+^ and AKI^−^ groups. In contrast to sCr, urinary L-FABP concentrations in the AKI^+^ group showed rapid increase, as early as 2 h after receiving CIS-CT, and were significantly higher by Hour 6 than in the AKI^−^ group. This high concentration persisted until Day 2, then decreased again rapidly; the two groups no longer significantly differed in urinary L-FABP concentration after Day 3. However, urinary L-FABP concentrations improved to baseline in the AKI^−^ group, but did not return to baseline in the AKI^+^ group.

**Fig. 3:**
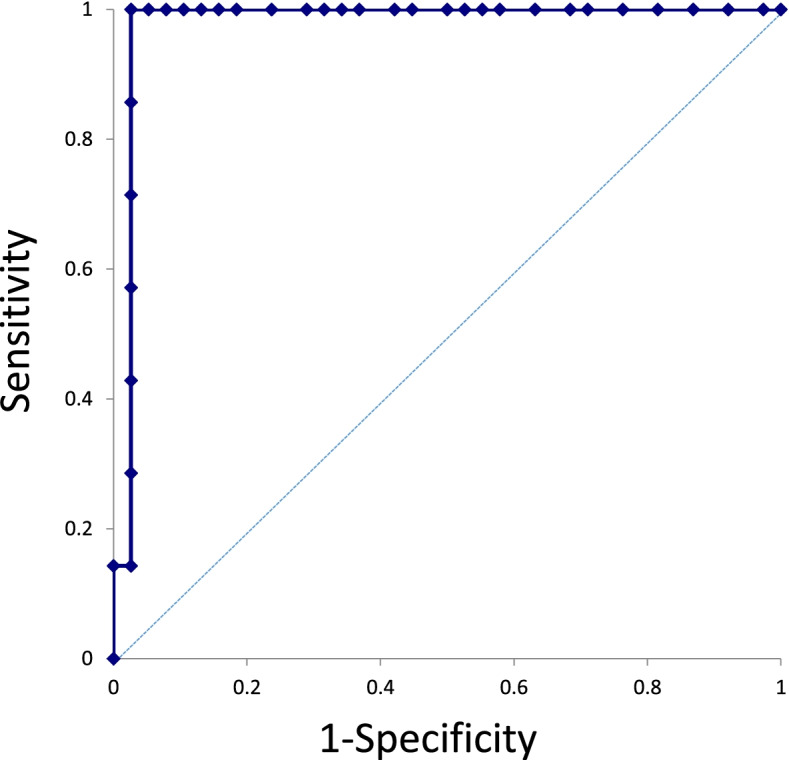
ROC analysis was performed to evaluate the superiority of urinary L-FABP magnification which had the highest at the hour 6

ROC analysis was performed to evaluate the superiority of urinary L-FABP magnification which had the highest at the hour 6 (Fig. 3). The urinary L-FABP magnification and levels of aria under curve was 0.977. Based on ROC analysis, the best cut-off value of urinary L-FABP magnification was 10.28 times urinary L-FABP levels at the hour 0 (base line urinary L-FABP).

## Discussion

In this study, despite hyperhydration and forced diuresis with furosemide and mannitol, 10 out of 42 patients who underwent CIS-CT to treat urothelial carcinoma suffered AKI. Moreover, acute renal function deterioration was predicted by increased urinary L-FABP excretion within 6 h after receiving CIS-CT and, in those with AKI, the increase in urinary L-FABP excretion preceded the rise in sCr by over 2 days. In contrast, no appreciable changes in urinary L-FABP levels were observed in patients with stable renal function throughout the whole observation period.

The weaknesses of using serum creatinine as a diagnostic marker for AKI include its relatively late elevation, its indirect relationship to kidney damage and its influence by extrarenal factors [15]. Differences in creatinine production related to age, sex, race, and weight, its secretion by the renal tubular epithelium, possible influence by some drugs, compromised metabolism of creatinine in AKI owing to hypercatabolism, and creatinine dilution in cases of volume overload are reasons why creatinine might not be a reliable marker for AKI. More sensitive and specific markers for acute renal injury are needed.

Recently identified urinary biomarkers for AKI include N-acetyl-D-glucosaminidase, α1-microglobulin, β2-microglobulin, NGAL, L-FABP, and KIM-1. NGAL [16] and Kim-1 [17] are reportedly useful for early detection of cisplatin-induced AKI. Although NGAL can be a useful marker for AKI [18, 19], urinary NGAL may be produced extrarenally in response to systemic stress; and urinary KIM-1 levels decrease significantly at lower and higher storage pH [20]. This means that some samples may have stability issues. Moreover, if urine cannot be immediately tested, accuracy may vary. In contrast, urinary L-FABP is not affected by urinary sediment and can therefore be measured in spot urine samples. Furthermore, urinary L-FABP measurements are highly reproducible [21]. Finally, rather than fluctuating with exercise [22], urinary L-FABP concentration is extremely stable. All these reasons indicate that urinary L-FABP may be a more stable and reproducible marker for evaluating early renal dysfunction.

In this study, more women were in the AKI^+^ group than in the AKI^−^ group. Risk factors for AKI included age > 65 years, female sex and metabolic complications in one report [23], and age > 70 years, diabetes mellitus, chronic kidney disease, heart disease, gout, infections, surgery and some drugs (NSAIDs, diuretics, aminoglycosides and vancomycin) in another report [24]. Other than sex, background factors, such as age, renal function and complications, did not significantly differ between the AKI^+^ and AKI^−^ groups in this study. Patients with upper tract urothelial carcinoma are often elderly (i.e., over 65 years of age) and often have only a single kidney owing to nephroureterectomy, so they are at higher risk of developing AKI. Therefore, early diagnosis of AKI is important for patients with upper tract urothelial carcinoma. As urinary L-FABP may help diagnose AKI within 6 h after cisplatin administration, it is a useful marker.

This study had some limitations. First, it was a single-centre, cross-sectional study with a small sample population. Second, the study group included only Japanese patients, which may limit generalizability of its results to other groups. Third, the eGFR averaged over 60 in this study. Different results may be obtained in patients with lower renal function.

In conclusion, early increase in urinary L-FABP may help identify patients at risk of cisplatin-induced AKI, who might benefit from treatments to prevent nephrotoxicity.

## Data Availability

The datasets during and/or analysed during the current study available from the corresponding author on reasonable request.

## Abbreviations

CIS-CT: Cisplatin-based chemotherapy
AKI: Acute kidney injury
eGFR: Estimated glomerular filtration rate
NGAL: Neutrophil gelatinase-associated lipocalin
IL-18: Interleukin-18
KIM-1: Kidney injury molecule-1
L-FABP: L-type fatty acidbinding proteins
KDIGO: Kidney Disease: Improving Global Outcomes
NAG: N-acetyl-D-glucosaminidase
